# Shrinkage, Permeation and Freeze–Thaw Characteristics of Ambient Cured High Calcium-Based Alkali-Activated Engineered Composites

**DOI:** 10.3390/ma16227101

**Published:** 2023-11-09

**Authors:** Khandaker M. A. Hossain, Dhruv Sood

**Affiliations:** Department of Civil Engineering, Toronto Metropolitan University, Toronto, ON M5B 2K3, Canada; dhruvsood@torontomu.ca

**Keywords:** alkali-activated engineered composite, industrial wastes, fiber, powder form reagents, shrinkage/permeation/freeze–thaw durability, microstructure

## Abstract

Sustainable zero cement-based one-part ambient cured alkali-activated engineered composites (AAECs) are developed. The durability and microstructural characteristics of developed AAECs using 2% *v*/*v* polyvinyl alcohol (PVA) fibers, silica sand, binary or ternary combinations of precursors (fly ash class C ‘FA-C’, fly ash class F ‘FA-F’ and ground granulated blast furnace slag ‘GGBFS’) and two types of powder form alkaline reagents (Type 1 and Type 2) are evaluated compared to conventional engineered cementitious composites (ECCs) and alkali-activated mortars (AAMs) without fiber. AAECs developed satisfactory compressive strength ranging from 34 MPa to 46 MPa. Expansion/shrinkage and mass change (loss/gain) behaviors are affected by binary/ternary combination of source materials, reagent types and curing regimes (water or ambient) for both AAMs and AAECs. The binary (FA-C + GGBFS) and reagent 2 (calcium hydroxide + sodium sulfate) composites demonstrated lower shrinkage due to formation of crystalline C-A-S-H/C-S-H binding phases than their ternary (FA-C + FA-F + GGBFS) and reagent 1 (calcium hydroxide + sodium metasilicate) counterparts which formed amorphous N-C-A-S-H/N-A-S-H phases. The matrix densification due to the formation of reaction products and fiber-induced micro-confinement leads to lower shrinkage and mass change of AAECs compared to their AAM counterparts. Composites exhibited lower or comparable secondary sorptivity indices compared to control ECC, indicating their superior permeation performance. All AAECs had a relative dynamic modulus of elasticity (RDME) greater than 90% at 300 cycles (comparable to control ECC), exhibiting satisfactory freeze–thaw resistance with reagent 2 mixes showing better performance compared to those with reagent 1. The production feasibility of strain hardening AAECs with powder form reagents having satisfactory mechanical and durability properties is confirmed.

## 1. Introduction

Durability and mechanical performance are the essential requisites for applying zero cement-based alkali-activated geopolymer materials incorporating industrial/agricultural wastes (such as fly ash, slag, rice husk ash, palm oil fuel ash, bagasse ash, recycled materials, etc.) as precursors in green sustainable construction [1,2,3,4,5,6]. The design parameters (reagent ratio, reagent molarity, etc.) of such materials and curing regimes had been related to their mechanical and durability properties [7]. An investigation was conducted on the effect of water-to-binder ratio, fundamental chemical ratios (SiO_2_/Al_2_O_3_ and Na_2_O/Al_2_O_3_) and the presence of ions (Na^+^ and K^+^) in the pore solution of the paste on the shrinkage with time [7]. It was found that Na^+^ ions improved the overall dimensional stability of the pastes [7]. Puertas et al. [8] investigated the shrinkage behavior and residual strength of polypropylene fiber-reinforced geopolymer composites (FRGC) subjected to freeze–thaw and wet-dry cycles. Among different source materials, fly ash (FA)-based FRGCs showed the lowest shrinkage at all ages under both humid (RH > 95%) and dry (RH = 50% in the laboratory) conditions. Both slag and slag/fly ash-based FRGC exhibited similar shrinkage compared to cement-based fiber-reinforced cementitious composite (FRCC) under dry laboratory conditions. The stability of the formed 3D alumino-silicate hydrate main reaction products was related to the superior performance of fly ash-based FRGC [8]. The dilution effect of fly ash on slag (fly ash:slag = 40:30) was also reported in fiber-reinforced engineered cementitious composites (ECCs) with strain hardening and micro-cracking characteristics having intrinsic crack width [9]. The refinement of pore structure due to the synergistic effect of fly ash slag led to the lowest shrinkage strains [9]. The dilution effect caused by the incorporation of fly ash into the ground granulated blast furnace slag (GGBFS) mix (50% replacement) was noted in another study, resulting in a reduction of shrinkage strains due to a lower degree of reaction [10]. The influence of incorporating basalt fiber (0, 10%, 20%, 30%, 40% and 100%) into the fly ash-based geopolymer paste on drying shrinkage was assessed [11]. It was observed that the total porosity, pore size and corresponding shrinkage of the geopolymer paste reduced with an increase in the percentage of basalt fiber, making it denser and more compact [11]. Shrinkage characteristics of natural pozzolana-based geopolymer paste with and without fiber were investigated [12]. An investigation of various curing methods at an early age on alkali-activated slag concrete (AASC) revealed that water curing decreased the drying shrinkage compared to air curing [13]. In addition, CO_2_ curing and water curing at elevated temperatures were beneficial to mitigate drying shrinkage. Heat curing decreased the drying shrinkage by up to 80%. This was attributed to the accelerated hydration-induced denser microstructure and consequent porosity reduction. Also, carbonation during CO_2_ curing led to the decalcification of C-A-S-H gel, coarsened pore size distribution and decreased total porosity. 

Freeze–thaw cycles generate internal stress, destroy pore structure, generate micro-cracks, enlarge pore diameter and increase pore connectivity that result in higher permeability and subsequent concrete deterioration due to higher ingress or diffusion of harmful agents in the matrix [14,15]. The influence of freezing and thawing on the mechanical behavior of FRGCs was also evaluated [8]. The slag-based FRGC exhibited good performance, showing 28% and 17% increases compared to 41% and 20% reduction by their fly ash-based counterparts in flexural and compressive strengths, respectively, after 50 freeze–thaw cycles. The cement-based FRCC also exhibited a decrease in flexural and compressive strengths after the freeze-thaw cycles [8]. The effect of fiber incorporation on the mechanical and durability aspects of one-part alkali-activated (sodium metasilicate) GGBFS-based binders was investigated [16]. The addition of fibers (in combinations or single) resulted in more reduction of compressive and flexural strengths after freeze–thaw cycles due to the increased presence of a connected void system and capillary network compared to an un-reinforced matrix. The effect of fiber on flexural strength loss was more significant than the compressive strength loss after freeze–thaw cycles. Incorporating polyvinyl alcohol fiber (PVA) and steel fibers into the matrix resulted in a more refined pore structure than the matrix with cellulose and basalt fibers having disconnected larger pores with lesser tortuosity. The addition of fibers to the matrix led to a 35% to 55% increase in apparent porosity when used in combinations (hybrid composite) and up to 65% when used as a single type of fiber compared to the control un-reinforced mortar. However, the PVA fibers were able to restrict flexural strength loss to 1%. Contrary to the compressive strength and flexural strength characteristics, the presence of the fibers led to lower mass loss than the unreinforced matrix. The type (steel, PVA, cellulose and basalt) of fibers had no substantial impact on the ultrasonic pulse velocity (UPV) or the air voids with reference to the un-reinforced matrix. Maximum variation of ±2% was observed in the UPV for various composites [16]. 

The metakaolin-incorporated slag-based alkali-activated materials can reduce shrinkage and improve durability due to the generation of a cross-linked C–A–S–H and N–A–S–H gel structures which decrease the content of bound water and pore water absorption [17]. The influence of nanoparticles on the freeze–thaw resistance of alkali-activated slag concrete was investigated [18]. The water permeation properties of alkali-activated binders (AABs) incorporating different pozzolans and Na-based activators were investigated at 90, 180, and 270 days [19]. AABs absorbed more water than those with ordinary Portland cement (OPC), especially at an early age, but reduced over later ages. The higher water absorption and lower strength of AABs lead to a higher sorptivity [19]. 

Existing research suggests limited studies on the durability and microstructure of strain-hardening ambient cured alkali-activated engineered composites (AAECs), especially using high calcium-based precursors, powder form reagents, micro-fine silica sand and PVA fibers. This research addressed these gaps by investigating the durability of such developed AAECs with the characterization of microstructural reaction products. The influences of various variables (such as combinations/proportions of reagents and binary/ternary combination of high calcium-based precursors) on the shrinkage/expansion of mortars (without PVA fibers)/composites (with PVA fibers) in two curing regimes (water/ambient), permeation properties (water absorption rate/sorptivity) and freeze–thaw resistance have been investigated as novel aspects of the study. The durability properties of AAECs incorporating high calcium reagents and precursors are compared with those of control cement-based ECC to evaluate comparative performance. The recommendations will facilitate engineers/researchers in understanding the durability characteristics of AAECs.

## 2. Experimental Investigations 

The experimental program consisted of the development and performance evaluation of strain-hardening AAECs in addition to comparing performance amongst alkali-activated mortars (AAMs) without PVA fibers, AAECs, control ECC and control cementitious mortar. The developed composites were assessed based on their durability (shrinkage, sorptivity and resistance to freeze–thaw cycles) and microstructural characteristics.

### 2.1. Materials and Mix Designs 

The mix proportions of eight alkali-activated mortars (AAMs), eight AAECs, a control cement mortar (FPcM) and an ECC are presented in Table 1. High calcium fly ash class C (FA-C), low calcium fly ash class F (FA-F), GGBFS, two powder-based reagents (reagent 1 and 2) and constant dosage (0.3 by mass of binder content) of silica sand (maximum particle size 600 µm) were used to develop AAMs as per authors’ research works [20]. AAEC mixes were made by using 2% *v*/*v* PVA fibers to the AAMs [21]. Detailed physical and chemical properties of all the materials can be found in [20].

Reagent 1 was composed of a combination of calcium hydroxide (Ca(OH)_2_) and sodium meta-silicate (Na_2_SiO_3_·5H_2_O) in the ratio (Ca(OH)_2_:Na_2_SiO_3_·5H_2_O) of 1:2.5. The constituents of the reagent 2 were calcium hydroxide (Ca(OH)_2_) and sodium sulfate (Na_2_SO_4_) in the ratio (Ca(OH)_2_:Na_2_SO_4_) of 2.5:1. Type GU-general use cement and FA-F were used for producing control ECC [22]. The dosage of high-range water-reducing admixture (HRWRA) was kept constant at 0.02 by mass of the binder in the alkali-activated composites while water-to-binder ratio ranged between 0.35 to 0.375 to achieve a mini-slump flow of at least 150 mm. Oil-coated 8 mm long PVA fibers were used with properties as shown in Table 1. The chemical ratios of mixes (Table 2) fall within the range of fly ash and slag-based alkali-activated materials [5,6]. The dosage of high-range water-reducing admixture (HRWRA) was kept constant at 0.006 mass of the binder for FPcM and ECC. AAEC mixes were produced from optimized mortars [20] developed through comprehensive experimental investigations. 

### 2.2. Mixing Protocols

The powdered reagents and precursors were rigorously blended separately before being dry-mixed together for about 5 min in a shear mixer. Then, two-thirds of the required water was gradually added and mixing continued to make a flowable paste for sand addition as per Table 1. Mixing continued with the gradual addition of remaining HRWRA mixed water to make the AAMs. PVA fibers were then added gradually to ensure full dispersion to produce AAECs, and the total mixing time ranged from 20 to 25 min. 

### 2.3. Test Methods

Three 50 mm × 50 mm × 50 mm cube specimens per mix composition were cast, de-molded 24 h after casting, kept in the curing room at 23 ± 2 °C and 95 ± 5% relative humidity (RH) and tested at 28 days for compressive strength following ASTM C109/C109M-16 [23]. 

Eight 25 mm × 25 mm × 285 mm prismatic specimens per mix composition were cast and kept in air-tight plastic bags until being de-molded after 24 h. The length and mass change for four specimens were taken at 1/7/28/56/90 days in the water curing regime as per ASTM C157/C157M-17 [24]. The other four specimens were cured in water for 28 days and then ambient/dry cured in a curing room (kept at an RH of 50 ± 4% and 23 ± 2 °C) with length/mass change recorded at 1/7/28/56/90 days. 

The water permeation properties defined based on sorptivity indices were determined as per ASTM C1585-13 [25] using 100 mm × 50 mm discs cut from 100 mm × 200 mm cylinders at 39 days (21 days of sealed curing and 18 days of sample preparation) as described in [18]. The initial and secondary sorptivity indices were calculated from the recorded mass change of the specimens at standard time intervals during testing. 

A freeze–thaw test was conducted on three 50.8 mm × 76.2 mm × 355.6 mm prism specimens for each composite mix sealed-cured for 14 days before being subjected to 300 cycles following ASTM C666/C666M-15 [26]. The mass change and ultrasonic pulse velocity (UPV) were measured at various cycles. Relative dynamic modulus of elasticity (RDME) in the transverse direction (width = 50.8 mm) was calculated as the % ratio of fundamental transverse frequency after specific cycles to that of 0 cycles [18,23]. 

SEM/EDS analysis of the chip specimens taken out from the cube samples was conducted to characterize the reaction products formed in the matrix. Detailed procedures of sample preparation and SEM/EDS analyses can be found in [21].

## 3. Results and Discussions

### 3.1. Compressive Strength

The compressive strength of AAECs at 28 days ranged between 34 MPa and 46 MPa compared to 52 MPa of conventional cement-based ECC, as presented in Table 3. PVA fiber addition to AAECs and ECC slightly increased their compressive strength compared to their AAMs (ranging between 34 and 42 MPa) and cementitious mortar (FPcM) counterparts without fiber (Table 3). Detailed compressive and flexural strengths of all these mixes can be found in [21].

### 3.2. Shrinkage/Expansion Characteristics

Table 3 presents the length change of the mortar and composite specimens in water curing at 56/90 days.

**Figure 1:**
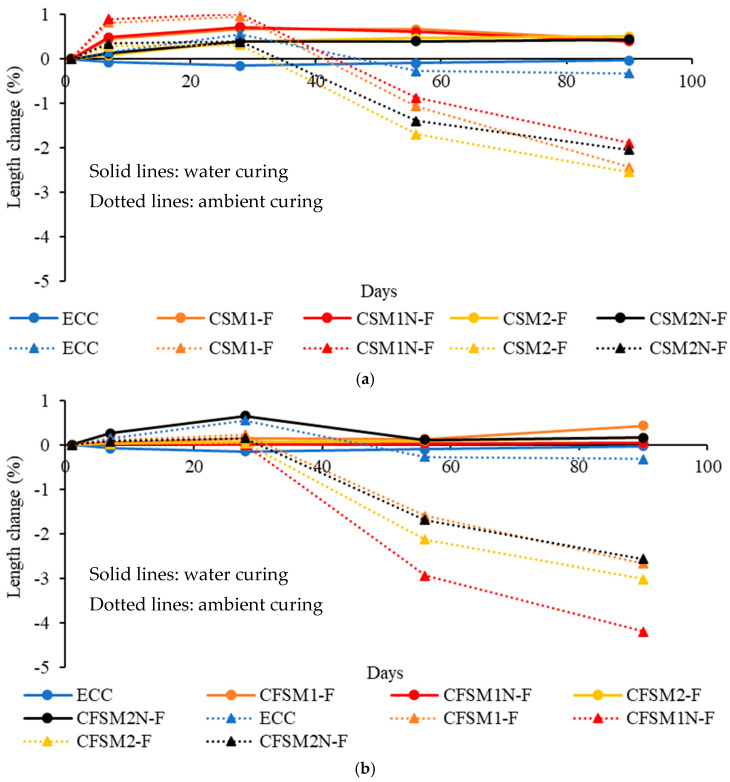
(**a**) Effect of binary blends of precursors on length change of composites. (**b**) Effect of ternary blends of precursors on length change of composites.

All the AAEC specimens observed expansion (+values) at all ages in water immersion, as evident from Figure 1a,b while ECC showed shrinkage (−values). The densification of the matrix by forming reaction products on the uniformly distributed fibers resulted in a more compact microstructure than their mortar systems. The active formation of reaction products can be concluded from the steeper length change (expansion) during the first seven days. The length change (expansion) seemed to remain the same after 56 days for all composites, showing completion of cementitious and geopolymer/alkali activation reaction product formation.

The specimens with binary composite mixes incorporating reagent 1 observed higher expansion strains with the highest value of 0.724% at 28 days for CSM1N-F than their counterparts with reagent 2 in water immersion, as indicated in Figure 1a,b. The combined effect of cementitious reaction products (as evident from SEM/EDS analysis discussed later) consuming water for hydration and the higher creep coefficient of C-A-S-H gels lead to more expansion/swelling. On the other hand, the appearance of additional C-S-H gel in composites with reagent 2 (CSM2-F and CSM2N-F) densified the microstructure (as confirmed by SEM/EDS analysis discussed later) and limited the expansive strains. Higher expansion strains were generally noted for binary composites than their ternary counterparts, as shown in Figure 1a,b and Table 3. This can be attributed to the primary formation of cementitious C-A-S-H/C-S-H binding phases, as discussed later in SEM/EDS analysis. On the other hand, a combination of cementitious (C-A-S-H/C-S-H) and amorphous (N-C-A-S-H/N-A-S-H) binding phases was observed in ternary composites (as observed in SEM/EDS analysis discussed later). The formation of cementitious reaction products consumed water for hydration, and the formation of amorphous products resulted in the release of water. Also, some FA-F remained unreacted in ternary composites and acted as a filler, reducing expansion strains. Control cementitious mortar (FPcM) showed expansion during water curing, while no definite trend of expansion or shrinkage was observed for AAM specimens (Table 3). 

The AAEC specimens started exhibiting shrinkage (−values) on getting shifted from water curing to ambient/air curing regime at 28 days (Figure 1a,b and Table 4). This could be attributed to the ongoing alkali activation resulting in the release of water that evaporated in drying or air curing conditions. The length and mass change of specimens made with mortars and composites in the ambient/air curing regime at 56/90 days are presented in Table 4 for comparative purposes. The composite specimens showed lesser variation in shrinkage strains than their mortar counterparts at 56 days in the ambient curing regime because of the micro-confinement created by the fibers in composites, as indicated in Table 4. The variation in length change (shrinkage) ranged from −0.874% to −2.94% for composite and from −0.032% to −2.82% for mortar specimens, respectively, at 56 days. All composite/mortar/control specimens exhibited shrinkage during the whole ambient curing regime after 28 days (Figure 1a,b and Table 4). 

The ternary composite specimens showed higher shrinkage strains than their binary counterparts in ambient curing (generally shrinkage increased with age, Table 4), as apparent from Figure 1a,b. The maximum length change of up to −1.7% (shrinkage) was observed for binary composite CSM2-F and up to −2.94% (shrinkage) for ternary composite CFSM1N-F at 56 days, as indicated in Table 4 for ambient curing. The higher shrinkage strains for ternary composites can be attributed to the larger formation of amorphous reaction products compared to the dominant formation of crystalline products in their binary counterparts. Similar behavior was noted in previous investigations reporting that the development of cementitious crystalline gels (C-A-S-H/C-S-H), as observed in this study, leads to lower shrinkage strains compared to the development of amorphous binding phases [27,28]. The production of these cementitious binding phases led to the refinement of the pore structure at the micro-level by the re-arrangement of capillary pores, similar to the observation reported in previous research [11]. In general, expansion/shrinkage behavior was affected by binary/ternary combination of source materials, reagent types and curing regimes for both AAMs and AAECs. 

### 3.3. Mass Change Characteristics 

**Figure 2:**
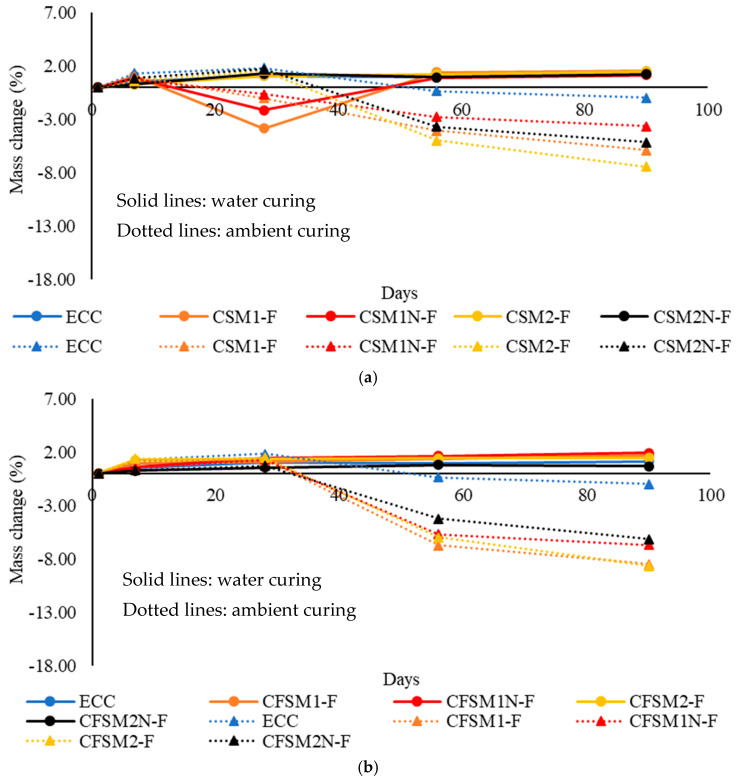
(**a**) Effect of binary blends of precursors on mass change of composites. (**b**) Effect of ternary blends of precursors on mass change of composites.

The mass change of the mortar and composite specimens in the water curing regime at 56/90 days are compared in Table 3. The mass change with time of the composite specimens in water curing is shown in Figure 2a,b. After 28 days, mass loss or increase was noticed for binary composites (Figure 2a) while a trend of increase (Figure 2b) was noticed for ternary mixes under water curing. However, continuous water curing led to mass gain and ambient curing after 28 days led to mass loss of AAEC specimens. An abrupt change (up to −3.86%) in mass was observed for binary composites incorporating reagent 1 (CSM1-F and CSM1N-F) at 28 days (Figure 2a). AAMs/AAECs/control mortar FPcM/ECC showed a trend of mass gain at later ages (56d and 90d) under continuous water curing (Table 3) and a trend of mass loss under ambient curing after 28 days (Table 4). 

However, the mass decline (loss) in specimens made of binary AAMs with reagent 1 was less compared to their counterpart AAECs in ambient curing. This can be attributed to the additional porosity created because of the incorporation of fibers. These fine-connected pores in composites lose water in dry conditions, and a greater mass change is observed compared to their counterpart mortar mixes. Similar behavior was noted in previous investigations where up to a 65% increase in apparent porosity was noted when the matrix was reinforced with fibers [16]. AAECs with reagent 2 demonstrated a positive mass change (gain) at all ages to the mass on the first day, as evident from Figure 2a,b under water curing. This can be associated with the development of dominant cementitious reaction products in these composites with reagent 2. Almost no mass change under continuous water curing after 56 days for all composite mixes might be indicative of the end of the reaction processes, as noted in Figure 2a,b. 

A slightly lower mass change for the composite specimens than their mortar counterparts in ambient/air curing conditions at 56 days was observed. A mass change of up to −6.73% and −9.56% was observed at 56 days for composite and mortar specimens, respectively, as per Table 4. Lower mass change in composites can be attributed to the densification of the reinforced matrix due to the formation of additional reaction products (both crystalline and amorphous) with time on the fibers acting as nucleation sites. The variation in mass for reagent 2 composites was lower than those with reagent 1 (CSM1-F, CSM1N-F, CFSM1-F and CFSM1N-F). This could be associated with the development of uniform reaction products consisting of crystalline cementitious gels in composites incorporating reagent 2. In contrast, a combination of amorphous geopolymer and crystalline cementitious gels composed the binding phases in composites with reagent 1. The uniformity in reaction products for composites with reagent 2 was responsible for fewer variations in mass change with age, as shown in Figure 2a,b. The ternary composites exhibited more mass change/decline up to −6.93% compared to −4.98% mass reduction in binary composites at 56 days under ambient curing, as noted in Table 4. The dominant formation of geopolymer reaction products in ternary composites is responsible for greater mass loss due to water released during geopolymerization evaporating in drying conditions. 

In general, the development of cementitious phases (C-A-S-H/C-S-H) in binary and reagent 2 composites led to lower shrinkage strains compared to their ternary and reagent 1 counterparts which showed production of dominant amorphous phases (N-C-A-S-H/N-A-S-H). Also, all AAECs and AAMs exhibited comparable shrinkage/expansion to the control mortar and ECC in the water curing regime. However, these alkali-activated mixes demonstrated higher shrinkage strains than the control cement-based mixes when the specimens were shifted to an air/ambient curing regime at 28 days due to different reaction processes involving the release of water in alkali activation compared to water utilization for cement hydration. Both AAMs and AAECs developed in this study showed comparable performance in shrinkage/expansion to the supplementary cementing materials (SCMs) or natural pozzolan-based geopolymers developed in previous studies [7,12,29] exhibiting shrinkage strains varying from 0.5% to 3.25%. Based on the experimental data, no relationship between length and mass change of composites can be established. 

### 3.4. Permeation (Water Absorption/Sorptivity) Characteristics 

**Figure 3:**
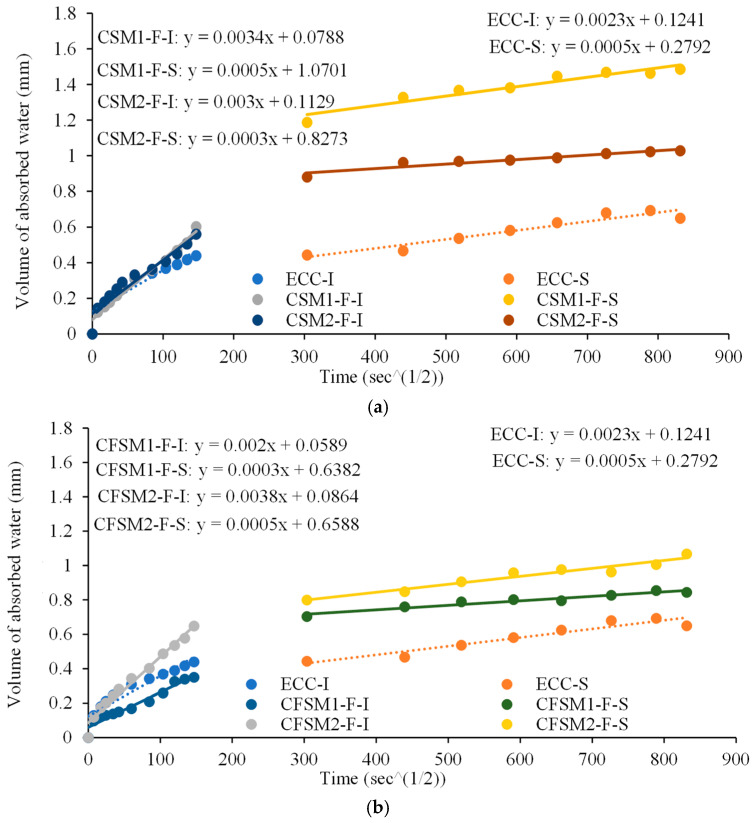
(**a**) Sorptivity indices for binary composites. (**b**) Sorptivity indices for ternary composites.

The linear relationship between water absorption and square root of time for binary and ternary composites showing initial (I) and secondary (S) sorptivity indices as slopes are shown in Figure 3a,b with root mean square values above 0.98. The sorptivity indices of all the mortars and composites are also compared in Table 5. The initial sorptivity indices of binary and ternary composite specimens ranged from 3.0 × 10^−3^ mm/$\sqrt{s}$ to 3.6 × 10^−3^ mm/$\sqrt{s}$ and 2.0 × 10^−3^ mm/$\sqrt{s}$ to 4.1 × 10^−3^ mm/$\sqrt{s}$, respectively. The N-designated composites with an equal mass of total FA and GGBFS showed slightly higher initial sorptivity indices than their counterpart mixes having, 40% to 45% GGBFS content. The higher GGBFS/calcium content in those mixes led to the predominant production of C-S-H/C-A-S-H, occupying fewer void spaces than the amorphous N-C-A-S-H/N-A-S-H. A similar observation was made for mortar mixes, as reported in Table 5. 

The initial sorptivity indices were 21% to 100% higher in composites than their mortar counterparts. Also, the mass change of composite specimens in shrinkage/expansion was higher along with more expansion/swelling in the water curing regime than their mortar counterparts and is consistent with previous studies [13,16,20]. The ongoing reaction process and the additional void spaces due to the presence of the fibers resulted in higher initial sorptivity indices of the composites. The presence of FA-F and the dominant formation of amorphous binding phases for ternary mixes with reagent 1 led to 39% to 41% lower initial and 40% lower secondary sorptivity indices than their binary counterparts. Also, these ternary mixes with reagent 1 exhibited up to 46% lower initial and 40% lower secondary sorptivity indices than their counterparts with reagent 2. Un-hydrated FA-F particles and the amorphous reaction products reduced the number of connected voids, which resulted in a lower capillary rise and permeability of the ternary composites with reagent 1. 

All the AAECs and AAMs except CFSM1 and CFSM1N exhibited higher reductions (between 74.5% and 93.9%) from primary to secondary sorptivity indices compared to the control mortar (FP_C_M) and ECC mixes (72.6% and 78.3% respectively), as noted in Table 5. Also, these AAECs and AAMs showed lower or comparable secondary sorptivity indices compared to control cementitious ECC and mortar (FP_C_M), indicating their superior water permeation performance. This can be associated with the formation of compact and less porous microstructure due to the formation of C-A-S-H/ C-S-H/N-C-A-S-H gels. Previous research involving alkali-activated fly ash/slag (AAFS) concretes also indicated slag content and the water-to-binder ratio as dominant factors to provide better permeation properties due to denser microstructure with reduced porosity leading to higher chloride penetration and corrosion resistance [30]. 

### 3.5. Resistance to Freezing and Thawing

Table 6 presents UPV values of the specimens at 0, 30, 60, 120 and 300 cycles. The RDME values were calculated from UPV values in the transverse direction. At 0 cycles, the UPV of composites varied from 3228 m/s to 3997 m/s and from 2361 m/s to 4047 m/s in longitudinal and transverse directions, respectively. Initially up to 60 cycles, UPV values showed an increasing trend for some mixes in both longitudinal/transverse directions and then showed a definite decreasing trend at a higher number of cycles of up to 300, exhibiting progressive freeze–thaw deterioration of specimens (Table 6). 

**Figure 4:**
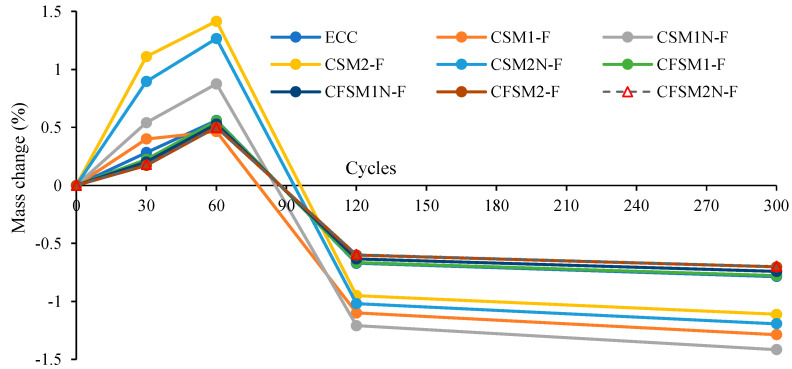
Mass change of composites with freeze–thaw cycles.

**Figure 5:**
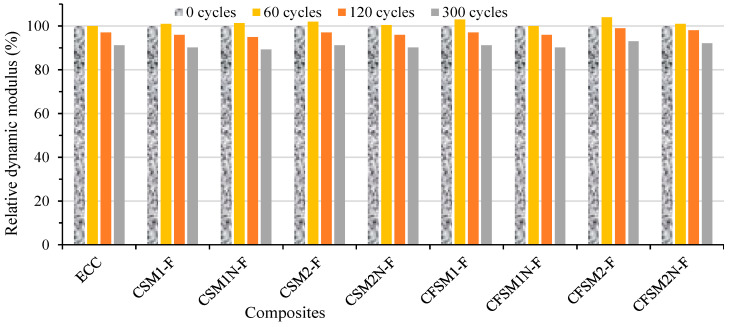
Variation of relative dynamic modulus of elasticity with freeze–thaw cycles.

The variation in mass change (loss or grain) and RDME values in percentage for composites (ECC and AAECCs) are shown in Figure 4 and Figure 5, respectively. A mass increase (maximum up to 1.41%) was observed for all composites up to 60 cycles (Figure 4) with RDME values of ≥100% showing no freeze–thaw deterioration (Figure 5). The improvement in RDME/UPV and corresponding initial mass gain (+value) up to 60 freeze–thaw cycles can be associated with more compact and refinement of microstructure due to changes in pore size and distribution. Beyond 60 cycles, a decreasing trend in mass change led to mass loss (Figure 4) associated with a decreasing trend of RDME values (Figure 5) exhibiting freeze–thaw deterioration, which was supported by the decreasing trend of UPV (Table 6). In general, binary mixes showed higher mass loss (−1.11 to −1.42%) and lower RDME values (89% to 91%) compared to ternary mixes which had −0.70% to −0.78% mass loss and 90% to 93% RDME. On the other hand, AAEC mixes with reagent 1 showed higher mass loss and lower RDME values compared to reagent 2 mixes. Ternary AAEC mixes and mixes with reagent 2 exhibited comparatively better freeze–thaw resistance performance. 

The lower RDME values of binary composites can also be related to their higher initial and secondary sorptivity indices compared to their ternary counterparts (Table 5). The reduction in UPV and RDME values was more predominant in reagent 1 binary composites than in their reagent 2 counterparts. Reagent 1 mixes showed lower UPV (Table 6) and lower RDME than those with reagent 2 (Figure 5) due to dominant production of C-S-H/C-A-S-H which made them denser and less prone to freeze–thaw damage as noted in their mortar counterparts. This is consistent with a previous study, where superior mortar compactness led to better freeze–thaw durability [18,20]. 

Ternary composites (CFSM1-F, CFSM1N-F, CFSM2-F and CFSM2N-F) performed better than their binary counterparts in terms of mass change (loss), UPV and RDME values. All composites exhibited 90% or more RDME (91% observed for control ECC) values having low mass loss (0.7% to 1.4%) with no sign of physical deterioration or spalling after 300 cycles, which indicated their satisfactory freeze–thaw resistance comparable to or better than ECC. 

### 3.6. Microstructural Analysis 

**Figure 6:**
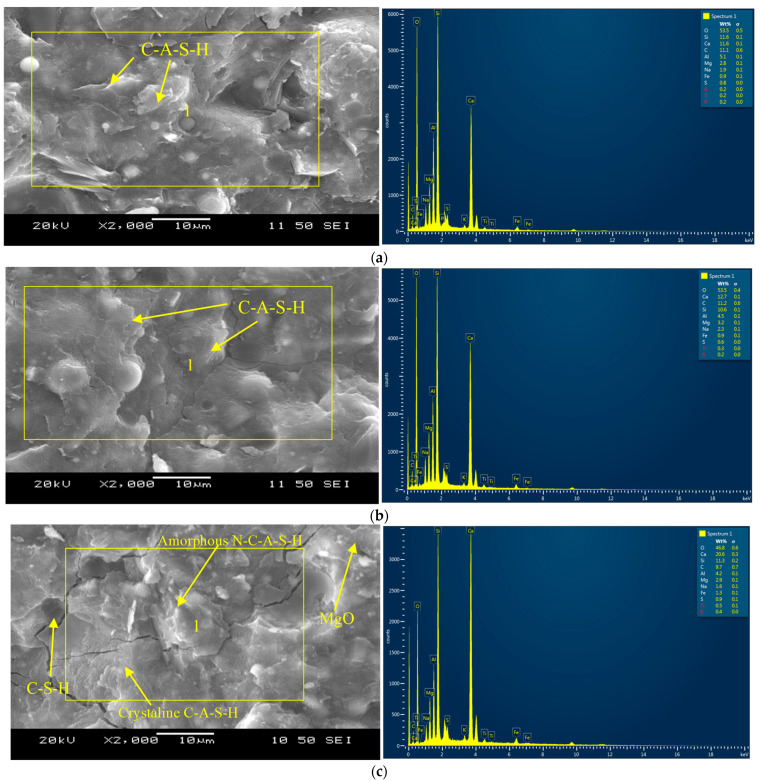

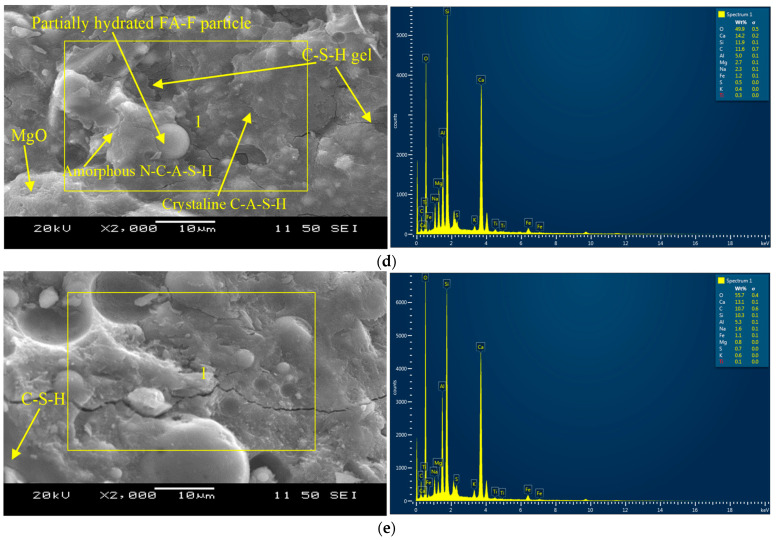
(**a**) SEM and EDS analyses–mortar CSM1. (**b**) SEM and EDS analyses–mortar CSM2. (**c**) SEM and EDS analyses–mortar CFSM1. (**d**) SEM and EDS analyses–mortar CFSM2. (**e**) SEM and EDS analyses–mortar FP_C_M.

Figure 6a–e shows the SEM/EDS graphs for mortar compositions, indicating the reaction products or the binding gels at 365 days. Both binary mortar mixes (CSM1 and CSM2) demonstrated similar elemental composition (Ca = 11.6–12.7%, Si = 10.6–11.6%, Al = 4.5–5.1% and O = 53.5%), as noted in the SEM/EDS in Figure 6a,b, indicating primary binding phase consisting of C-A-S-H. 

The ternary composition (CFSM2) appears to be denser than CFSM1 (Figure 6c,d) due to a 20% higher CaO/SiO_2_ ratio, which led to the additional C-S-H gel formation along with the C-A-S-H/N-C-A-S-H primary binding phases. Traces of periclase formation are also noticed in both ternary mixes. The major elements determined from the EDS analysis of ternary mortars are Ca = 14.2–20.6%, Si = 11.3–11.9%, Al = 4.2–5.0%, Mg = 2.7–2.9%, Na = 1.6–2.3% and O = 46.8–49.9% (Figure 6c,d). 

In general, reagent 2 composites/mortars performed better than their reagent 1 counterparts in terms of mechanical and durability characteristics due to the development of denser calcium-dominated reaction products with traces of C-S-H gels (Figure 6b,d), as explained earlier. 

C-S-H gel was observed as the primary reaction product for the control cement-based mortar (FP_C_M) as inferred by the elemental compositions (Ca =13.1%, Si =10.3%, Al = 5.3% and O = 55.7%) in the EDS pictograph presented in Figure 6e. Whereas for AAMs, amorphous N-C-A-S-H and crystalline C-A-S-H and C-S-H were identified from SEM/EDS analysis presented in Figure 6a–d.

**Figure 7:**
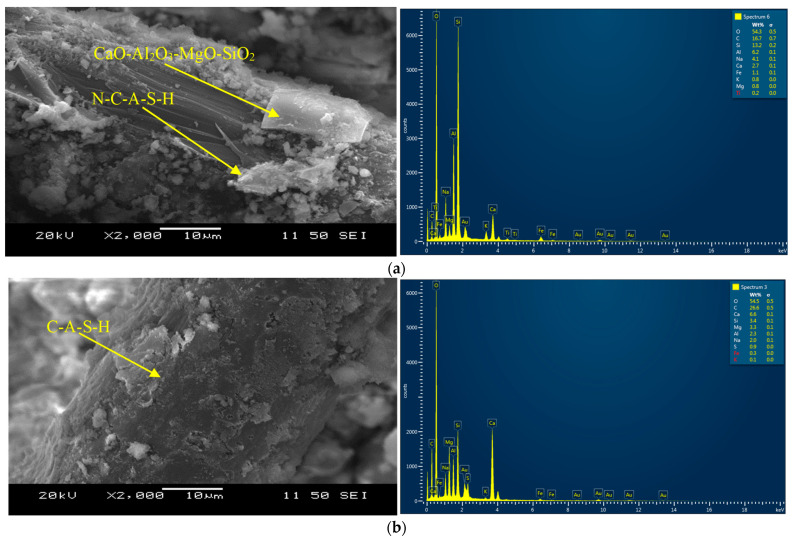
(**a**): SEM and EDS analyses-composite CFSM1-F at 28 days. (**b**): SEM and EDS analyses–composite CFSM2-F at 28 days.

Figure 7a,b presents the binding phases produced on the PVA fiber in ternary composites CFSM1-F and CFSM2-F. The glassy texture on the fiber indicates the development of phases CaO-Al_2_O_3_-MgO-SiO_2_ consisting of silica–alumina linkages (Si = 13.2%, Al = 6.2%, O = 54.3%) with the oxygen atom as noted in EDS analysis of CFSM1-F shown in Figure 7a. A denser layer of crystalline C-S-H/C-A-S-H compared to that of composite CFSM1-F is formed on the fiber embedded in ternary CFSM2-F with traces of N-C-A-S-H/N-A-S-H as shown in Figure 7b. The formation of traces of gypsum, as noticed from the EDS pictogram, indicated ettringite formation that led to further micro-structure densification similar to that observed in a study on FA-F and GGBFS-incorporated mixes with combined calcium hydroxide-sodium sulfate activators [31]. Strong PVA fiber-reaction product bond (an indication of efficient fiber-matrix interaction), is evident from the SEM micrographs (Figure 7a,b) with a major binding phase composed of C-A-S-H/N-C-A-S-H as observed for their mortar counterparts. GGBFS-incorporated mortars also developed similar binding phases in previous research [32]. 

## 4. Conclusions

The following conclusions were drawn from this study:

- The compressive strength of AAECs at 28 days ranged between 34 MPa and 46 MPa (>18 MPa) compared to 52 MPa of conventional cement-based ECC, meeting the criteria for structural applications. PVA fiber addition to AAECs and ECC slightly increased their compressive strength compared to their AAMs without fiber (ranging between 34 and 42 MPa).
- The binary AAECs (made of FA-C and GGBFS) demonstrated lower shrinkage strains (up to −1.77%) and mass decrease (up to −4.98%) in ambient/air curing regime compared to their ternary (made of FA-C, FA-F and GGBFS) counterparts exhibiting shrinkage strains and mass decrease of up to −2.94% and −6.93%, respectively at 56 days. This was attributed to the formation of densified C-A-S-H with additional C-S-H, which led to more compact and denser binary composites than their ternary counterparts. The densification of AAECs due to reaction product development on fibers and fiber-induced micro-confinement leads to lower shrinkage strains and mass change in ambient curing conditions compared to their AAM counterparts.
- The additional porosity/pore network created by the fibers resulted in higher initial sorptivity indices in AAECs than in AAMs. Reagent 1 (calcium hydroxide:sodium silicate = 2.5:1) based ternary AAECs exhibited the lowest initial sorptivity indices (from 0.002 to 0.0022 mm/$\sqrt{s}$) amongst all mixes, as some of the partially/un-hydrated fly ash class-F particles acted as fillers reducing pore sizes and number of connected voids. AAECs and AAMs showed lower or comparable secondary sorptivity indices compared to the control ECC and cementitious mortar (FP_C_M).
- All AAMs and AAECs exhibited higher shrinkage strains than the control ECC mix but showed comparable shrinkage characteristics to the existing alkali-activated materials in the literature. This was attributed to the different reaction processes occurring in the alkali-activated systems and the cement-based materials, namely conventional control ECC.
- All AAECs exhibited satisfactory freeze–thaw resistance, showing relative dynamic modulus of elasticity (RDME) of greater than 90% at 300 cycles with reagent 2 mixes showing higher RDME values compared to those with reagent 1.
- SEM/EDS analysis revealed possible chemical reaction/bonding between the PVA fibers and the reaction products (cementitious and silica-alumina-rich/alkali-activated gels). Efficient bonding between the fibers and reaction products/gels was evident from the SEM micrographs.
- Developed cement-free AAECs showed comparable or superior performance compared to their control cementitious ECC counterparts in terms of strength and durability characteristics, demonstrating their potential applications in sustainable constructions. However, binary AAECs with reagent 1 are preferable in terms of better compressive strength and shrinkage characteristics, similar other properties and simpler mix compositions.

Investigations are in progress to study the durability of AAECs under fire and chemical composures, in addition to introducing multi-functional properties such as self-healing and self-sensing through the incorporation of carbon nanomaterials and healing agents. 

## Figures and Tables

**Table 1:** Mix proportions for AAMs and AAECs.

Mix ID (Mortars, Composites)	Total SCMs (Binder *)	Portland Cement (Pc)	FA-C	FA-F	GGBFS	Reagent Type	Reagent/ Binder Ratio	Silica Sand	Water-to-Binder Ratio
AAM and PVA fiber incorporated AAEC (with end letter F in mix ID) mixes; CS: Binary and CFS: Ternary mixes									
CSM1, CSM1-F	1	0	0.55	0	0.45	1	0.09	0.3	0.35
CSM1N, CSM1N-F	1	0	0.50	0	0.50	1	0.09	0.3	0.35
CFSM1, CFSM1-F	1	0	0.25	0.35	0.40	1	0.09	0.3	0.35
CFSM1N, CFSM1N-F	1	0	0.25	0.25	0.50	1	0.09	0.3	0.35
CSM2, CSM2-F	1	0	0.55	0	0.45	2	0.12	0.3	0.375
CSM2N, CSM2N-F	1	0	0.50	0	0.50	2	0.12	0.3	0.375
CFSM2, CFSM2-F	1	0	0.25	0.35	0.40	2	0.12	0.3	0.375
CFSM2N, CFSM2N-F	1	0	0.25	0.25	0.50	2	0.12	0.3	0.375
Control mortar and PVA fiber incorporated engineered cementitious composite (ECC) mixes									
FP_C_M/ECC	1	0.45	0	0.55	0	-	-	0.36	0.27

Numeric in mix proportions represents mass ratio of binder; Precursors: C: FA-C, F: FA-F, S: GGBFS. In mix ID: N indicates mixes with equal mass of fly ash (class C+ class F) and GGBFS; F denotes PVA fiber incorporated AAEC mixes, the numeric value denotes reagent type, M denotes mix 2% (*v*/*v*) PVA fibers were used in AAECs and control ECC PVA fiber: diameter: 38 µm, elastic modulus = 41 GPa, density = 1.3 g/cm^3^), tensile strength = 1610 MPa. * Binder: precursors + Portland cement (P_C_).

**Table 2:** Reagent and mix parameters.

Mix ID (Mortars, Composites)	Reagent		Chemical Ratios (Precursors + Reagents)			
	Type	Component Ratio	SiO_2_/Al_2_O_3_	Na_2_O/SiO_2_	CaO/SiO_2_	Na_2_O/Al_2_O_3_
CSM1, CSM1-F	1	1:2.5	2.62	0.09	0.84	0.23
CSM1N, CSM1N-F	1	1:2.5	2.71	0.08	0.87	0.23
CFSM1, CFSM1-F	1	1:2.5	2.75	0.08	0.59	0.22
CFSM1N, CFSM1N-F	1	1:2.5	2.86	0.07	0.69	0.21
CSM2, CSM2-F	2	2.5:1	2.56	0.14	1.02	0.35
CSM2N, CSM2N-F	2	2.5:1	2.64	0.13	1.02	0.35
CFSM2, CFSM2-F	2	2.5:1	2.69	0.12	0.73	0.32
CFSM2N, CFSM2N-F	2	2.5:1	2.80	0.12	0.84	0.33
FP_C_M, ECC	-	-	2.70	0.06	0.82	0.16

**Table 3:** Strength and change in length/mass of mortars and composites under water curing.

Control Mortar/ AAMs	Comp. Strength (MPa)	Length Change (%)		Mass Change (%)		ECC/AAECs	Comp. Strength (MPa)	Length Change (%)		Mass Change (%)	
	28 d	56 d	90 d	56 d	90 d		28 d	56 d	90 d	56 d	90 d
FP_C_M	43.5	0.59	0.55	1.69	1.75	ECC	52.5	−0.10	−0.03	0.94	1.16
CSM1	42.6	−0.58	−0.41	0.22	0.48	CSM1-F	46.5	0.67	0.43	1.42	1.52
CSM1N	35.0	−0.56	−0.35	0.23	0.54	CSM1N-F	34.1	0.61	0.40	0.92	1.15
CSM2	41.2	0.35	0.45	1.86	2.41	CSM2-F	41.2	0.47	0.51	1.23	1.48
CSM2N	35.8	−0.15	−0.15	0.71	0.80	CSM2N-F	40.8	0.40	0.44	0.96	1.24
CFSM1	40.4	−0.39	−0.22	0.69	1.24	CFSM1-F	44.3	0.14	0.43	1.37	1.70
CFSM1N	34	−0.26	−0.18	0.99	0.85	CFSM1N-F	36.5	0.04	0.05	1.65	1.92
CFSM2	42	0.13	0.32	0.64	0.55	CFSM2-F	42	0.10	0.16	1.45	1.48
CFSM2N	38.1	0.13	0.29	1.70	1.35	CFSM2N-F	39.2	0.11	0.16	0.79	0.66

Negative indicates shrinkage and mass loss, and positive values denotes expansion and mass gain; AAMs: alkali activated mortars, AAECs: alkali activated engineered composites, ECC: engineered cementitious composite.

**Table 4:** Change in length and mass of mortars and composites under ambient curing.

Control Mortar/AAMs	Length Change (%)		Mass Change (%)		ECC/AAECs	Length Change (%)		Mass Change (%)	
	56 d	90 d	56 d	90 d		56 d	90 d	56 d	90 d
FP_C_M	−0.04	−0.22	−0.08	−0.52	ECC	−0.27	−0.32	−0.36	−0.99
CSM1	−1.33	−1.34	−7.51	−11.28	CSM1-F	−1.06	−2.44	−4.06	−5.90
CSM1N	−0.96	−0.90	−5.56	−7.36	CSM1N-F	−0.87	−1.89	−2.76	−3.65
CSM2	−1.98	−2.65	−6.15	−8.21	CSM2-F	−1.70	−2.55	−4.98	−7.44
CSM2N	−1.58	−2.14	−6.71	−8.67	CSM2N-F	−1.40	−2.04	−3.69	−5.16
CFSM1	−2.82	−3.46	−9.56	−10.60	CFSM1-F	−1.60	−2.66	−6.73	−8.49
CFSM1N	−2.61	−4.14	−6.66	−8.38	CFSM1N-F	−2.94	−4.20	−5.70	−6.69
CFSM2	−1.62	−2.82	−5.73	−9.60	CFSM2-F	−2.13	−3.02	−6.01	−8.67
CFSM2N	−0.03	−1.66	−0.86	−3.51	CFSM2N-F	−1.69	−2.57	−4.24	−6.15

Negative values indicate shrinkage and mass loss, and positive values denote expansion and mass gain; AAMs: alkali-activated mortars, AAECs: alkali-activated engineered composites, ECC: engineered cementitious composites.

**Table 5:** Sorptivity indices for mortars and composites.

Mix. Des.	Mortars			Composites		
Mortars, Composites	Initial (I) Sorptivity (mm/$\sqrt{s}$)	Secondary (S) Sorptivity (mm/$\sqrt{s}$)	Decrease from I to S Sorptivity (%)	Initial Sorptivity (mm/$\sqrt{s}$)	Secondary Sorptivity (mm/$\sqrt{s}$)	Decrease from I to S Sorptivity (%)
FP_C_M, ECC	1.9 × 10^−3^	5.2 × 10^−4^	72.6	2.3 × 10^−3^	5.0 × 10^−4^	78.3
CSM1, CSM1-F	2.0 × 10^−3^	5.1 × 10^−4^	74.5	3.4 × 10^−3^	5.3 × 10^−4^	84.4
CSM1N, CSM1N-F	2.3 × 10^−3^	5.3 × 10^−4^	77.0	3.6 × 10^−3^	4.9 × 10^−4^	86.4
CSM2, CSM2-F	4.9 × 10^−3^	4.4 × 10^−4^	91.0	3.0 × 10^−3^	2.5 × 10^−4^	91.7
CSM2N, CSM2N-F	5.1 × 10^−3^	4.5 × 10^−4^	91.2	3.2 × 10^−3^	3.2 × 10^−4^	90.0
CFSM1, CFSM1-F	1.0 × 10^−3^	3.1 × 10^−4^	69.0	2 × 10^−3^	2.6 × 10^−4^	87.0
CFSM1N, CFSM1N-F	1.1 × 10^−3^	3.4 × 10^−4^	69.1	2.2 × 10^−3^	3.3 × 10^−4^	85.0
CFSM2, CFSM2-F	3.0 × 10^−3^	1.9 × 10^−4^	93.7	3.8 × 10^−3^	4.6 × 10^−4^	87.9
CFSM2N, CFSM2N-F	3.3 × 10^−3^	2.0 × 10^−4^	93.9	4.1 × 10^−3^	4.7 × 10^−4^	88.5

**Table 6:** UPV values of composites at various freeze–thaw cycles.

Composites	Ultrasonic Pulse Velocity (UPV) in Longitudinal Direction (355.6 mm)					Ultrasonic Pulse Velocity (UPV) in Transverse Direction (50.8 mm)				
	Number of Freeze–Thaw Cycles									
	0	30	60	120	300	0	30	60	120	300
ECC	3977	4022	4000	3800	3640	4047	4080	4047	3804	3642
CSM1-F	3358	3243	3600	3420	3276	3227	2318	3493	3283	3144
CSM1N-F	3532	3330	3471	3297	3159	3517	2849	3541	3329	3187
CSM2-F	3614	3515	3571	3392	3250	3617	3617	3493	3283	3144
CSM2N-F	3947	3887	3912	3716	3560	3984	4014	3922	3687	3530
CFSM1-F	3389	3368	3441	3269	3131	2428	2428	2550	2397	2295
CFSM1N-F	3425	3409	3351	3183	3049	3493	3517	3493	3283	3144
CFSM2-F	3228	3214	3138	2981	2856	2361	2318	2394	2250	2155
CFSM2N-F	3636	3600	3448	3276	3138	3777	3722	3750	3525	3375

## Data Availability

The data presented in this study are available on request from the corresponding author.

## Abbreviations

AAEC	alkali-activated engineered composite
AAM	alkali-activated mortar
PVA	polyvinyl alcohol
FA-C	fly ash class C
FA-F	fly ash class F
GGBFS	ground granulated blast furnace slag
ECC	engineered cementitious composite
Pc	Portland cement
FPcM	cement mortar
UPV	ultrasonic pulse velocity
RDM	relative dynamic modulus of elasticity
SEM	scanning electron microcopy
EDS	energy dispersive spectroscopy
